# Circulating calprotectin (S100A8/A9) is higher in rheumatoid arthritis patients that relapse within 12 months of tapering anti-rheumatic drugs

**DOI:** 10.1186/s13075-019-2064-y

**Published:** 2019-12-05

**Authors:** Emma C. de Moel, Jürgen Rech, Michael Mahler, Johannes Roth, Thomas Vogl, Anne Schouffoer, Robbert J. Goekoop, Tom W. J. Huizinga, Cornelia F. Allaart, René E. M. Toes, Georg Schett, Diane van der Woude

**Affiliations:** 1 Department of Rheumatology, Leiden University Medical Center, Leiden, the Netherlands; 2 Department of Internal Medicine 3, Rheumatology and Immunology, Friedrich-Alexander University of Erlangen-Nuremberg and Universitatsklinikum Erlangen, Erlangen, Germany; 3 Inova Diagnostics, San Diego, CA USA; 4 Institute of Immunology, University of Muenster, Muenster, Germany; 5 Department of Rheumatology, Haga Ziekenhuis, The Hague, the Netherlands

**Keywords:** Calprotectin, S100A8/A9, MRP8/14, Rheumatoid arthritis, Disease activity

## Abstract

**Objective:**

To investigate whether calprotectin (S100A8/A9 or MRP8/14), an inflammatory complex released by monocytes, could indicate residual subclinical inflammation in rheumatoid arthritis (RA) patients who are in stable remission on disease-modifying anti-rheumatic drugs (DMARDs) and serve as a marker for disease flare after DMARD tapering.

**Methods:**

We used data from two trials. Patients from the IMPROVED study had early (< 2 years) RA, and when they achieved disease activity score remission (DAS44 < 1.6), they stopped methotrexate to attempt drug-free remission. Patients from the RETRO study had established RA in stable remission (DAS28 < 2.6) and either tapered by 50% or stopped (biological or conventional) DMARDs. Circulating calprotectin at the tapering time point was determined by ELISA, and its predictive value for flare (loss of remission) within 12 months of DMARD tapering/stopping was determined.

**Results:**

In both IMPROVED (*n* = 104) and RETRO (*n* = 57), patients that flared within 12 months had higher calprotectin at the moment of DMARD tapering/stopping. Twofold higher calprotectin at the moment of DMARD tapering/stopping was associated with an increased risk (odds ratio) of flare of 1.07 (95% CI 0.98–1.18, *p* = 0.14) in the IMPROVED and 3.62 (95% CI 1.76–7.46, *p* < 0.001) in the RETRO. Correcting for clinical predictors of flare (DAS at study inclusion, anti-CCP2 positivity, gender) did not change these estimates. The area under the receiver operating curve of calprotectin levels for predicting flare within 12 months was 0.63 (95% CIs 0.51–0.76) in the IMPROVED study and 0.80 (95% CIs 0.69 to 0.92) in the RETRO study.

**Conclusion:**

Circulating calprotectin levels in RA patients in remission on DMARDs are higher in patients that will flare upon DMARD tapering/stopping. Since the differences between the cohorts precluded definitive conclusions, more research is needed to determine whether calprotectin has prognostic value in predicting flare after attempting drug tapering in RA.

**Trial registration:**

IMPROVED, ISRCTN11916566. RETRO, 2009-015740-42.

## Introduction

In rheumatoid arthritis (RA), improvements in the control of inflammation by conventional synthetic and biologic disease-modifying antirheumatic drugs (DMARDs) are slowly shifting therapeutic goals from preventing damage by suppressing disease activity to tapering DMARDs in patients in stable remission to achieve drug-free remission (DFR). However, DFR will not be reached by all RA patients in remission, and the disease flare that can occur after unsuccessful DMARD tapering can increase disease burden that could have been avoided if the DMARDs had not been interrupted. Hence, identifying factors associated with increased risk of disease flare after tapering DMARDs would aid clinical decision-making.

Unfortunately, this endeavor has proven difficult. Most studies agree that seropositivity [both rheumatoid factor (RF) and anti-cyclic citrullinated protein antibodies (ACPA)], shared epitope presence, high disease activity at baseline, and long symptom duration are risk factors for flaring upon DMARD tapering [1, 2]. Residual subclinical inflammation has also been proposed to be a major predictor of flare, prompting multiple studies to identify biomarkers sensitive in detecting residual inflammation at the moment of tapering, including multibiomarker disease activity scores [3] and inflammation on imaging [1]. However, these markers are limited by variable associations and lack of replication.

More recently, circulating calprotectin has gained traction as a promising biomarker for RA disease activity. Calprotectin, also called S100A8/A9 or myeloid-related protein (MRP) 8/14, is a heterodimeric complex of the S100 family constitutively expressed in leukocytes. During infectious and inflammatory events, calprotectin acts as alarmin and amplifies inflammation by binding to receptors for advanced glycation end-products and Toll-like receptor 4. Traditionally measured in feces in inflammatory bowel disease diagnostics, calprotectin differs from hepatocyte-dependent acute-phase reactants like C-reactive protein (CRP) and erythrocyte sedimentation rate (ESR) because it is locally released by leukocytes at the sites of inflammation [4]. In arthritis, activated granulocytes and tissue-resident macrophages in inflamed joints may contribute to calprotectin release. Various studies indicate that calprotectin actively promotes inflammation during arthritis [5, 6], is elevated in the synovial fluid and blood of RA patients, correlates consistently with markers of RA disease activity, is associated with ultrasound-detected inflammation, predicts future structural damage, and may have value in monitoring early response to biological DMARDs [7, 8]. Furthermore, in juvenile idiopathic arthritis (JIA), a high level of calprotectin correlated with a high risk of relapse after etanercept [9] and methotrexate [10] discontinuation. The same was true in systemic-onset JIA after anakinra discontinuation [11].

All of these findings point to the intimate link between calprotectin, neutrophil activation, and inflammation in arthritis. If calprotectin indeed indicates residual subclinical inflammation in RA patients who are in stable remission on DMARDs, it could serve as a practical biomarker for future disease flare after DMARD tapering and facilitate risk stratification to predict whether DMARD tapering will be successful. Such data could aid in clinical decision-making and replace the trial-and-error strategies by which DFR is currently—and infrequently—achieved. To that end, we investigated in two independent cohorts whether circulating calprotectin in patients who are in remission on DMARDs is associated with risk of disease flare after DMARD tapering and discontinuation.

## Methods

### Study design, patient population, and outcomes

We used data from two multicenter randomized controlled trials: the IMPROVED study (an acronym for Induction therapy with MTX and Prednisone in Rheumatoid Or Very Early arthritic Disease) and the RETRO study (an acronym for Study of Reduction of Therapy in Patients with Rheumatoid Arthritis in Ongoing Remission). The IMPROVED study [12] enrolled 610 patients with early (< 2 years) untreated RA or undifferentiated arthritis and was steered at disease activity score remission (DAS44 < 1.6). In patients achieving remission, DMARDs were stopped, aiming for drug-free remission (DFR), with treatment adjusted every 4 months according to whether remission was maintained. Initial treatment comprised MTX and high-dose prednisone. Subjects selected for this study were all 104 patients who (1) fulfilled the 2010 ACR/EULAR RA criteria, (2) achieved remission at 4 months and stopped MTX at 8 months, and (3) had serum available for calprotectin testing at 8 months.

The RETRO study [13] enrolled patients with established RA who had been in stable remission (DAS28-ESR ≤ 2.6) for at least 6 months on conventional and/or biological DMARD treatment regimen. Patients were randomized to three treatment arms: (1) continue DMARDs at full dose for 12 months, (2) taper current dose by 50% for the next 12 months, or (3) reduce current dose by 50% for the first 6 months before entirely stopping all DMARDs. All patients in the taper arm (arm 2, *n* = 33) or stop arm (arm 3, *n* = 24) were included for this analysis.

Autoantibody status of the patients from both studies was determined by routine clinical testing for anti-CCP2 (IgG) and RF (IgM). The main outcome for both studies was flare within 12 months of tapering/stopping DMARDs, defined in the IMPROVED as a DAS44 ≥ 1.6 determined at 4-month intervals and in the RETRO as DAS28-ESR > 2.6 determined at 3-month intervals.

### Calprotectin measurements

Patient serum was collected when patients were in remission and stored in − 80 °C until use. In the IMPROVED, circulating calprotectin was measured at baseline and 8 months (month of MTX stopping) using a fecal Calprotectin Extended Range kit adapted for serum (QUANTA Lite®, Inova Diagnostics, research use only for serum). In the RETRO, calprotectin was measured in baseline samples, which was at study inclusion directly preceding DMARD tapering, before any tapering or stopping changes were made to existing DMARDs, using an in-house sandwich ELISA developed by Johannes Roth (Muenster) for predicting flares in juvenile idiopathic arthritis as previously described [11]. Absorbance was converted to nanograms per milliliter using a standard curve of serial dilutions of a known concentration.

### Statistical analysis

Calprotectin levels (in ng/mL) were compared between the groups using Mann-Whitney *U* tests, and correlations with inflammatory parameters were explored cross-sectionally using Spearman tests. Univariable and multivariable binary logistic regression was performed with disease flare within 12 months as the outcome. Calprotectin levels were log2-transformed and included as a continuous covariate. Area under the receiver operating characteristic curves (AUCs), test characteristics, and predictive values of calprotectin levels for flare were calculated, and the optimum cutoff was determined using the Liu index [14].

Additionally, logistic regression models were used to evaluate whether the addition of calprotectin to a clinical predictor model for flare significantly improved prediction of flare within 12 months. Clinical predictors of flare with a *p* value of ≤ 0.1 in univariable models were considered for the multivariable models to discover clinical predictors common to both cohorts, as well as specific to each cohort (as a sensitivity analysis, see Additional file 1: Table S1A-B). Areas under the ROC curve were calculated from the predicted values of the model with and without calprotectin and compared using a chi-square-based test for equality [15]. All analyses were conducted separately for each cohort, using Stata SE 14.1.

## Results

### In patients in remission, calprotectin is not associated with inflammatory parameters or autoantibody status

The characteristics of both cohorts are displayed in Table 1.

**Table 1:** Patient characteristics

	IMPROVED (*N* = 104)	RETRO (*N* = 57)
Age, mean years (SD)	49 (13)	55 (13)
Female, *n* (%)	67 (64%)	37 (65%)
BMI, mean (SD)	25 (4)	25 (4)
Ever smoker, *n* (%)	47 (46%)	18 (32%)
Disease duration, median (IQR)	19 (9–36) weeks^^^	5 (3–10) years
Anti-CCP2 IgG positive, *n* (%)	85 (83%)	34 (62%)
RF IgM positive, *n* (%)	79 (79%)	39 (68%)
Current DMARD use^†^		
Methotrexate	104 (100%)	47 (82%)
Glucocorticoids	0 (0%)	11 (19%)
Other csDMARDS	0 (0%)	6^‡^ (11%)
Biological DMARDs	0 (0%)	21 (37%)
Etanercept	–	5 (9%)
Adalimumab	–	6 (11%)
Tocilizumab	–	5 (9%)
Golimumab	–	2 (4%)
Certolizumab	–	3 (5%)
DAS44 at tapering/stopping moment, mean (SD)	0.9 (0.4)	–
DAS28-ESR at tapering/stopping moment, mean (SD)	–	1.8 (0.7)
Disease flare within 12 months	78 (75%)	26 (46%)
Calprotectin (ng/mL), median (IQR)	1000 (230–2422)	1000 (650–2000)

*SD* standard deviation, *IQR* interquartile range, *Anti-CCP2* anti-citrullinated protein 2 antibody, *RF* rheumatoid factor, *cs/bDMARD* conventional synthetic/biological disease-modifying antirheumatic drugs, *DAS* disease activity score, *ESR* erythrocyte sedimentation rate

^^^Disease duration is based on the moment of study inclusion; in the IMPROVED study, this excludes the first 4 months of treatment

^†^Categories are not mutually exclusive

^‡^Patients received sulfasalazine (*n* = 2), hydroxychloroquine (*n* = 2), leflunomide (*n* = 1), or azathioprine (*n* = 1)

Circulating calprotectin levels, measured at the moment of DMARD tapering (for RETRO) or stop (for IMPROVED), were equal in the IMPROVED and the RETRO (*p* = 0.51), as well as between the two arms of the RETRO study (*p* = 0.43). Calprotectin levels were not correlated with acute-phase reactants (ESR, CRP), disease activity scores (DAS), or physical function as measured by the Health Assessment Questionnaire (HAQ) in either cohort, most likely due to the low variability of these parameters in these patients in remission (Additional file 1: Figure S1), and baseline demographics did not differ between patients with high vs. low calprotectin levels (Additional file 1: Table S2). In baseline serum of the IMPROVED study, when patients had active disease activity (DAS44 ≥ 2.6) and had not yet received DMARD treatment, calprotectin did correlate with these inflammatory parameters (not shown), in a similar magnitude to what has been reported [7].

Calprotectin levels were higher in seropositive than seronegative patients, although not significantly: median (IQR) levels in anti-CCP2 IgG-positive vs. anti-CCP2 IgG-negative patients were 1115 (309–2749) ng/mL vs. 764 (0–1636) ng/mL in IMPROVED (*p* = 0.21) and 1075 (800–2400) ng/mL vs. 930 (570–1850) ng/mL in RETRO (*p* = 0.45). In RF IgM-positive vs. RF IgM-negative patients, this was 1115 (237–2448) ng/mL vs. 982 (224–3318) ng/mL in IMPROVED (*p* = 0.96) and 1050 (550–1700) ng/mL vs. 965 (650–2300) ng/mL in RETRO (*p* = 0.75).

### Calprotectin levels are higher preceding flare than no flare

**Fig. 1:**
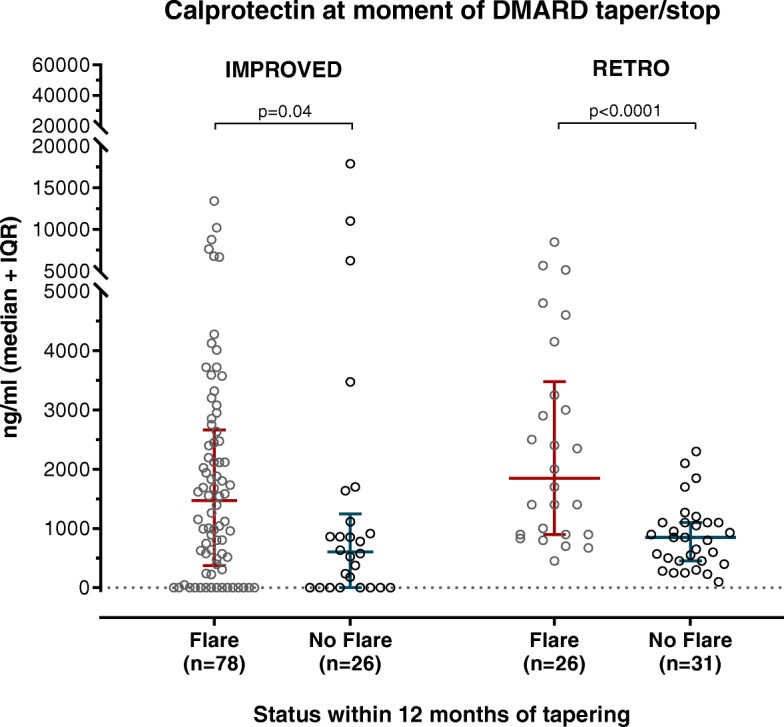
Circulating calprotectin levels (ng/mL) at the moment of DMARD tapering/stopping, separated by whether patients experienced a disease flare within 12 months of tapering. *p* values were calculated by Mann-Whitney *U* tests

In the IMPROVED study, disease flare was defined as DAS44 ≥ 1.6 within 12 months of stopping MTX. In the RETRO, flare was defined as DAS28-ESR > 2.6 within 12 months of tapering or stopping (biological or conventional) DMARDs. As indicated by Fig. 1, calprotectin was significantly higher at the moment of DMARD tapering/stopping in patients that experienced a disease flare within 12 months.

### Calprotectin levels are associated with disease flare

In a logistic regression model with log2-transformed calprotectin as a predictor, twofold higher calprotectin at the moment of DMARD tapering/stopping was associated with an increased risk (odds ratio) of flare of 1.07 (95% CI 0.98–1.18, *p* = 0.14) in the IMPROVED study and 3.62 (95% CI 1.76–7.46, *p* < 0.001) in the RETRO study. Correcting for clinical predictors of flare (DAS at study inclusion, anti-CCP2 positivity, gender) resulted in similar estimates: 1.09 (95% CI 0.98–1.21, *p* = 0.11) in the IMPROVED study and 3.77 (95% CI 1.74–8.18, *p* < 0.001) in the RETRO study. Correcting for clinical predictors of flare selected for their predictive value specific for each cohort (ESR at tapering moment, gender, anti-CCP2 positivity, and DAS at study inclusion in IMPROVED; SJC and anti-CCP2 positivity in RETRO) resulted in similar estimates (not shown).

**Fig. 2:**
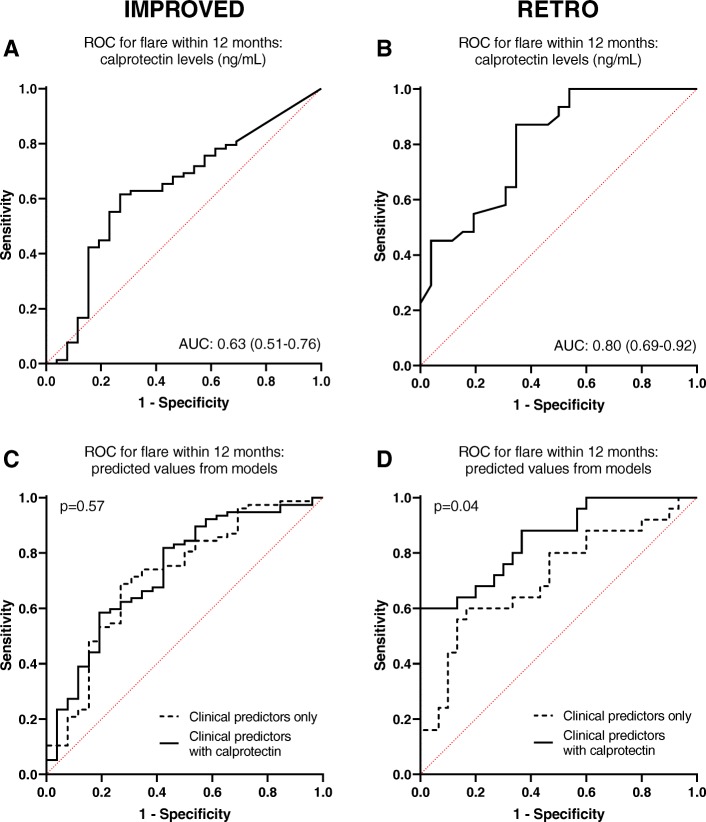
ROC curves indicating the predictive value of calprotectin and clinical predictors for disease flare within 12 months of tapering in the IMPROVED (**a**, **c**) and RETRO (**b**, **d**) studies. **a**, **b** Circulating calprotectin levels (ng/mL) at the moment of DMARD tapering/stopping. **c**, **d** Comparison of models including clinical predictors only (dashed line) and clinical predictors in combination with circulating calprotectin levels (solid line). The *p* value is based on the test for equality of AUCs

The AUC of calprotectin levels predicting flare in the IMPROVED study was 0.63 (95% CI 0.51–0.76, *p* = 0.002), indicating modest to poor discriminatory capacity (Fig. 2a). In the RETRO, calprotectin had a slightly better AUC of 0.80 (95% CI 0.69–0.92, *p* = 0.0001) for predicting flare within 12 months (Fig. 2b).

In the IMPROVED, addition of calprotectin levels did not significantly improve a model including only the clinical predictors (DAS as baseline, anti-CCP2 positivity, gender): the clinical predictor model had an AUC of 0.72 (95% CI 0.60–0.84), compared to a model combining calprotectin and clinical predictors that had an AUC of 0.73 (95% CI 0.62–0.85) (*p* for test of equality of AUCs = 0.57; Fig. 2c). However, in the RETRO, the addition of calprotectin to a clinical predictor model significantly improved the AUC from 0.71 (95% CI 0.57–0.85) to 0.85 (95% CI 0.75–0.95) (*p* for the test of equality of AUCs = 0.04; Fig. 2d). These results were the same when cohort-specific clinical predictors mentioned above were used (not shown).

### Test characteristics based on optimal and clinically relevant cutoffs

At an optimal cutoff determined using the Liu index, calprotectin levels above 936 ng/mL in the IMPROVED had the following test characteristics (95% CI) for disease flare within 12 months: 62% (50–72%) sensitivity, 73% (52–88%) specificity, 87% (76–95%) positive predictive value (PPV), and 39% (25–54%) negative predictive value (NPV). In the RETRO, calprotectin levels above 1335 ng/mL had slightly better test characteristics (95% CI): 65% (44–83%) sensitivity, 87% (70–96%) specificity, 81% (58–95%) PPV, and 75% (59–88%) NPV.

Since identifying patients with high flare risk that should not taper DMARDs is particularly relevant, we also examined a practical cutoff that maximized specificity. For the IMPROVED, calprotectin levels above 3571 ng/mL (corresponding to the 85th percentile) were 88% (70–98%) specific for flaring. In the RETRO, calprotectin levels above 2350 ng/mL (corresponding to the 79th percentile) were 100% (89–100%) specific for flaring. Patients classified as at risk for flare in this way (i.e., above this optimal cutoff) did not have higher inflammatory parameters (ESR, CRP, DAS, VAS, or HAQ) than those predicted not to flare (i.e., below the cutoff; not shown). In clinical practice, one could therefore infer from very high calprotectin levels that a patient is likely to flare regardless of other inflammatory parameters and should not taper DMARDs.

## Discussion

In this study, we evaluated the role of circulating calprotectin in RA as a predictor for disease flare upon DMARD tapering and discontinuation in two independent cohorts. Higher circulating calprotectin levels were associated with significantly increased risk of flare within 12 months after DMARD tapering in the RETRO study and a small, non-significant effect in the IMPROVED study. In the RETRO study, calprotectin significantly improved the identification of patients that would flare within 12 months over clinical predictors alone.

Calprotectin has been reported to correlate better with clinical disease activity of RA than acute-phase reactants do [16]. These findings suggest that calprotectin levels may reflect local inflammatory processes in the joints and therefore make it of particular value in predicting whether inflammation has been abrogated and patients can achieve drug-free remission. Possessing a marker that can accurately indicate residual inflammation in patients in remission would help to optimize therapeutic decision-making by accurately stratifying which patients should and should not attempt to taper DMARDs.

In line with the literature [16], circulating calprotectin levels in our study were variable and sometimes quite high, even in patients in remission, a disease state in which systemic inflammation is, in principle, low. Although we were not able to replicate previous findings regarding the correlation between calprotectin and joint indices due to the lack of variability in joint indices, the fact that calprotectin did not correlate with other inflammatory parameters in these patients in remission suggests that calprotectin may indeed indicate residual inflammation not measured by these parameters, probably because it is highly expressed and released specifically at local sites of inflammation [4].

However, calprotectin’s value for predicting disease flare was not consistent in both cohorts. Although the direction of the found effect was the same in both cohorts, the effect sizes we found are quite divergent and only the RETRO study reached consistent statistical significance, possibly due to the differences between the cohorts. One major difference is the use of DAS44 in the IMPROVED and DAS28-ESR in the RETRO, which does not detect affected joints in the feet and thus could miss peripheral disease activity in the feet. It also is conceivable that calprotectin is not sensitive enough to detect residual inflammation when DAS is extremely low, as was the case in the IMPROVED study (DAS44 < 1.6 instead of DAS28-ESR < 2.6 in RETRO). However, a sensitivity analysis restricting the analyses in the IMPROVED cohort only to patients on the higher side of the spectrum (DAS44 ≥ 0.9 at 8 months) did not indicate that calprotectin had more value in patients with slightly higher disease activity (not shown). It is also possible that calprotectin cannot predict minute changes in DAS (e.g., small flares), but a sensitivity analysis in the IMPROVED study in which DAS44 ≥ 2.6 within 12 months was regarded as flare (more comparable to the RETRO definition) did not indicate that calprotectin had greater value in predicting larger DAS44 change-based flares than smaller flares (not shown). Differences in the biological use, disease durations, and remission duration could explain the discrepant results as well. However, stratification on biological use in the RETRO resulted in very small numbers and inconclusive results, and the disease and remission durations between the cohorts were so divergent that stratification on this parameter would not yield fair comparisons.

Some previous studies investigating calprotectin and flares in rheumatic diseases other than RA support calprotectin as a marker for flare after tapering DMARDs [10, 11], but Tweehuysen et al. found no value of calprotectin for predicting successful DMARD tapering in RA [17]. The main difference between our cohorts and the DRESS study (an acronym for Dose REduction Strategy of Subcutaneous TNF inhibitors) they investigated was that all patients in their study tapered biological DMARDs, specifically TNFα inhibitors. It is possible that TNFα inhibitors may directly inhibit the release of calprotectin in vivo and dampen calprotectin’s value as a marker of local inflammation, thereby also limiting its ability to predict future disease flare. However, beyond the upregulation of calprotectin by TNFα in murine models [18, 19], an inverse correlation between trough serum TNFα inhibitor levels and circulating calprotectin [20], a consistent decrease of calprotectin upon TNFα inhibitor initiation [8], no studies to date have explored this possibility.

Surprisingly, the population of the RETRO study, in which the association of calprotectin with flare was most convincing, is more similar to the DRESS study (long-standing disease, biological use including TNFα inhibitors, higher DAS at tapering) than the IMPROVED population is, even though the latter two cohorts showed little to no association of calprotectin with flare. This makes it very difficult to speculate on the reasons why these three cohorts show such divergent results, as there is clearly no pattern regarding patient characteristics and strength of associations found. Because of this, our results cannot definitively refute or commend calprotectin as a marker for successful DMARD tapering.

A further limitation to consider is that calprotectin was determined using different assays, and although circulating concentrations were converted to nanograms per milliliter using a known concentration curve in both cohorts, small differences in the sensitivity of the assays could remain. Sample handling may also have differed between various centers participating in the studies. However, there was no difference in calprotectin levels between the cohorts on the group level, so the effect of these limitations is most likely minor.

Although our results show modest effects, they are important because they situate calprotectin in a new field of prognostication in RA—that of drug-free remission. Currently, there is no consensus regarding calprotectin’s use as a measure of disease activity or a biomarker for treatment response, let alone the possibility of using it as a marker of future disease flare, and more studies are needed to evaluate calprotectin in this role.

## Conclusion

In conclusion, we found that calprotectin levels are higher in RA patients that will flare upon DMARD tapering. More research is needed to validate calprotectin as a biomarker for flare in RA patients tapering or discontinuing DMARDs.

## Supplementary information

**Additional file 1: Figure S1.** Correlation of circulating calprotectin levels (ng/mL) with inflammatory parameters. **Table S1.** Model building for outcome flare. **Table S2.** Inflammatory parameters and baseline demographics separated by low/high circulating calprotectin levels (ng/mL).

## Data Availability

The datasets used and/or analyzed during the current study are available from the corresponding author upon reasonable request.

## References

[1] Schett G, Emery P, Tanaka Y, Burmester G, Pisetsky DS, Naredo E (2016). Tapering biologic and conventional DMARD therapy in rheumatoid arthritis: current evidence and future directions. Ann Rheum Dis.

[2] van den Broek M, Huizinga TW, Dijkmans BA, Allaart CF (2011). Drug-free remission: is it already possible?. Curr Opin Rheumatol.

[3] Rech J, Hueber AJ, Finzel S, Englbrecht M, Haschka J, Manger B (2016). Prediction of disease relapses by multibiomarker disease activity and autoantibody status in patients with rheumatoid arthritis on tapering DMARD treatment. Ann Rheum Dis.

[4] Vogl T, Eisenblatter M, Voller T, Zenker S, Hermann S, van Lent P (2014). Alarmin S100A8/S100A9 as a biomarker for molecular imaging of local inflammatory activity. Nat Commun.

[5] van Lent PL, Grevers L, Blom AB, Sloetjes A, Mort JS, Vogl T (2008). Myeloid-related proteins S100A8/S100A9 regulate joint inflammation and cartilage destruction during antigen-induced arthritis. Ann Rheum Dis.

[6] Vogl T, Stratis A, Wixler V, Voller T, Thurainayagam S, Jorch SK (2018). Autoinhibitory regulation of S100A8/S100A9 alarmin activity locally restricts sterile inflammation. J Clin Invest.

[7] Bae SC, Lee YH (2017). Calprotectin levels in rheumatoid arthritis and their correlation with disease activity: a meta-analysis. Postgrad Med.

[8] Abildtrup M, Kingsley GH, Scott DL (2015). Calprotectin as a biomarker for rheumatoid arthritis: a systematic review. J Rheumatol.

[9] Anink J, Van Suijlekom-Smit LW, Otten MH, Prince FH, van Rossum MA, Dolman KM (2015). MRP8/14 serum levels as a predictor of response to starting and stopping anti-TNF treatment in juvenile idiopathic arthritis. Arthritis Res Ther.

[10] Foell D, Wulffraat N, Wedderburn LR, Wittkowski H, Frosch M, Gerss J (2010). Methotrexate withdrawal at 6 vs 12 months in juvenile idiopathic arthritis in remission: a randomized clinical trial. Jama..

[11] Holzinger D, Frosch M, Kastrup A, Prince FH, Otten MH, Van Suijlekom-Smit LW (2012). The Toll-like receptor 4 agonist MRP8/14 protein complex is a sensitive indicator for disease activity and predicts relapses in systemic-onset juvenile idiopathic arthritis. Ann Rheum Dis.

[12] Wevers-de Boer K, Visser K, Heimans L, Ronday HK, Molenaar E, Groenendael JH (2012). Remission induction therapy with methotrexate and prednisone in patients with early rheumatoid and undifferentiated arthritis (the IMPROVED study). Ann Rheum Dis.

[13] Haschka J, Englbrecht M, Hueber AJ, Manger B, Kleyer A, Reiser M (2016). Relapse rates in patients with rheumatoid arthritis in stable remission tapering or stopping antirheumatic therapy: interim results from the prospective randomised controlled RETRO study. Ann Rheum Dis.

[14] Liu X (2012). Classification accuracy and cut point selection. Stat Med.

[15] DeLong ER, DeLong DM, Clarke-Pearson DL (1988). Comparing the areas under two or more correlated receiver operating characteristic curves: a nonparametric approach. Biometrics..

[16] Inciarte-Mundo J, Ruiz-Esquide V, Hernandez MV, Canete JD, Cabrera-Villalba SR, Ramirez J (2015). Calprotectin more accurately discriminates the disease status of rheumatoid arthritis patients receiving tocilizumab than acute phase reactants. Rheumatology..

[17] Tweehuysen L, den Broeder N, van Herwaarden N, Joosten LAB, van Lent PL, Vogl T (2018). Predictive value of serum calprotectin (S100A8/A9) for clinical response after starting or tapering anti-TNF treatment in patients with rheumatoid arthritis. RMD Open.

[18] Xu K, Geczy CL (2000). IFN-gamma and TNF regulate macrophage expression of the chemotactic S100 protein S100A8. J Immunol.

[19] Koenders MI, Marijnissen RJ, Devesa I, Lubberts E, Joosten LA, Roth J (2011). Tumor necrosis factor-interleukin-17 interplay induces S100A8, interleukin-1beta, and matrix metalloproteinases, and drives irreversible cartilage destruction in murine arthritis: rationale for combination treatment during arthritis. Arthritis Rheum.

[20] Inciarte-Mundo J, Ramirez J, Hernandez MV, Ruiz-Esquide V, Cuervo A, Cabrera-Villalba SR (2016). Calprotectin and TNF trough serum levels identify power Doppler ultrasound synovitis in rheumatoid arthritis and psoriatic arthritis patients in remission or with low disease activity. Arthritis Res Ther.

