# Supplementary material for: Circulating calprotectin (S100A8/A9) is higher in rheumatoid arthritis patients that relapse within 12 months of tapering anti-rheumatic drugs
Source: Arthritis Res Ther. 2019 Dec 5;21:268. doi: 10.1186/s13075-019-2064-y (PMC6894482; doi:10.1186/s13075-019-2064-y)
Supplement: Supplementary file 1 — Additional file 1: Figure S1. Correlation of circulating calprotectin levels (ng/mL) with inflammatory parameters. Table S1. Model building for outcome flare. Table S2. Inflammatory parameters and baseline demographics separated by low/high circulating calprotectin levels (ng/mL). [file 13075_2019_2064_MOESM1_ESM.docx]

| **Figure S1:** Circulating calprotectin levels (ng/mL) at the moment of DMARD tapering/stop does not correlate with inflammatory parameters in the IMPROVED (left) or the RETRO (right) study. VASgh = VAS global health. | |
| --- | --- |
|  |  |

**Table S1A**: Model building for outcome flare in the IMPROVED study. All variables were tested univariably for association with the outcome; only those variables with p≤0.1 were included in multivariable models. Multivariable models were tested separately for activity parameters (top) and for demographic/baseline variables (bottom).

| IMPROVED study (n=104) |  |  |  |  |  |  |
| --- | --- | --- | --- | --- | --- | --- |
| Activity parameters at  tapering/stop moment | **Flare (n=78)** | **No Flare (n=26)** | **Univariable OR  (95% CI)** | **p-value** | **Multivariable OR (95% CI)** | **p-value** |
| DAS44, mean (SD) | 0.9 (0.4) | 0.7 (0.4) | 3.67 (1.07-12.6) | **0.04** | 1.16 (0.21-6.44) | 0.87 |
| ESR, median (IQR) | 6 (3-14) | 6 (2-9) | 1.10 (1.01-1.19) | **0.04** | 1.10 (1.01-1.20) | **0.04** |
| CRP, median (IQR) | 3 (3-6) | 3 (1-3) | 1.01 (0.97-1.06) | 0.56 |  |  |
| VASgh, median (IQR) | 10 (2-26) | 4 (2-8) | 1.05 (1.00-1.09) | **0.03** | 1.03 (0.98-1.08) | 0.21 |
| Tender joint count-28, median (IQR), (minimum-maximum) | 0 (0-0), (0-4) | 0 (0-0), (0-4) | 1.02 (0.62-1.70) | 0.93 |  |  |
| Swollen joint count-28, median (IQR),  (minimum-maximum) | 0 (0-0), (0-3) | 0 (0-0), (0-1) | 1.84 (0.47-7.28) | 0.87 |  |  |
| HAQ, median (IQR) | 0 (0-0.38) | 0 (0-0.13) | 33.0 (1.53-711) | **0.03** | 18.8 (0.71-498) | 0.08 |
| Demographic/baseline variables | **Flare (n=78)** | **No Flare (n=26)** | **Univariable OR  (95% CI)** | **p-value** | **Multivariable OR (95% CI)** | **p-value** |
| Age, mean years (SD) | 49 (14) | 51 (10) | 0.99 (0.95-1.02) | 0.43 |  |  |
| Female, % | 69% | 50% | 2.25 (0.91-5.57) | **0.08** | 3.10 (1.11-8.66) | **0.03** |
| BMI, mean (SD) | 26 (4) | 25 (3) | 1.06 (0.92-1.22) | 0.41***** | 1.15 (0.97-1.36) | 0.11 |
| Ever smoker, % | 44% | 52% | 0.71 (0.29-1.76) | 0.46 |  |  |
| Disease duration, weeks median (IQR) | 19 (3-39) | 18 (11-26) | 1.02 (0.99-1.04) | 0.14 |  |  |
| Anti-CCP2 IgG positive, % | 84% | 77% | 1.63 (0.54-4.89) | 0.39***** | 3.47 (0.95-12.6) | **0.05** |
| RF IgM positive, % | 80% | 75% | 1.36 (0.46-4.00) | 0.58 |  |  |
| DAS44, mean (SD) | 3.1 (0.8) | 2.7 (0.9) | 2.11 (1.12-3.97) | **0.02** | 2.45 (1.24-4.85) | **0.01** |
| HAQ, median (IQR) | 1 (0.63-1.5) | 0.75 (0.38-1.13) | 1.95 (0.89-4.26) | 0.10 |  |  |

* Anti-CCP2 IgG positivity and BMI were noteworthy at p=0.07 and p=0.03 respectively as a likely predictors for flare in a screening model including all demographic/baseline variables together (not shown), and were therefore included in the subsequent multivariable model.

Final IMPROVED-specific multivariable model including ESR at tapering moment, gender, anti-CCP2 positivity, and DAS44 at baseline had a pseudo-R^2^ of 14%. Final model including predictors common to both cohorts (DAS at baseline, anti-CCP2 positivity, gender) had a pseudo R^2^ of 11% in the IMPROVED study. Choice of these common predictors was based on known predictors of flare reported in literature as well as commonalities in predictors for both cohorts, reported above.

**Table S1B**: Model building for outcome flare in the RETRO study. All variables were tested univariably for association with the outcome; only those variables with p≤0.1 were included in multivariable models. Multivariable models were tested separately for activity parameters (top) and for demographic/baseline variables (bottom).

| RETRO study (n=57) |  |  |  |  |  |  |
| --- | --- | --- | --- | --- | --- | --- |
| Activity parameters at  tapering/stop moment | **Flare (n=26)** | **No Flare (n=31)** | **Univariable OR (95% CI)** | **p-value** | **Multivariable OR (95% CI)** | **p-value** |
| DAS28-ESR at tapering/stop moment, mean (SD) | 1.8 (0.7) | 1.8 (0.8) | 1.02 (0.50-2.11) | 0.95 |  |  |
| ESR, median (IQR) | 13 (7-19) | 13 (8-18) | 1.00 (0.94-1.05) | 0.87 |  |  |
| CRP, median (IQR) | 32 (11-70) | 20 (13-34) | 0.94 (0.41-2.20) | 0.89 |  |  |
| VASgh, median (IQR) | 0 (0-5) | 0 (0-4) | 0.97 (0.90-10.5) | 0.47 |  |  |
| Tender joint count-28, median (IQR), (minimum-maximum) | 0 (0-0), (0-1) | 0 (0-0), (0-1) | 0.27 (0.03 -2.58) | 0.26 |  |  |
| Swollen joint count-28, median (IQR),  (minimum-maximum) | 0 (0-0), (0-2) | 0 (0-0), (0-1) | 6.27 (0.74-53.18) | **0.09** | **n.d.*** |  |
| Demographic/baseline variables | **Flare (n=26)** | **No Flare (n=31)** | **Univariable OR (95% CI)** | **p-value** | **Multivariable OR (95% CI)** | **p-value** |
| Age, mean years (SD) | 56 (13) | 55 (13) | 1.00 (0.96-1.04) | 0.98 |  |  |
| Female, % | 69% | 61% | 1.42 (0.47-4.28) | 0.62 |  |  |
| BMI, mean (SD) | 25 (3) | 26 (4) | 0.91 (0.79-1.05) | 0.22 |  |  |
| Ever smoker, % | 31% | 32% | 0.93 (0.30-2.87) | 0.90 |  |  |
| Disease duration, years median (IQR) | 8 (3-10) | 4 (2-8) | 1.04 (0.98-1.12) | 0.20 |  |  |
| Anti-CCP2 IgG positive, % | 80% | 47% | 4.57 (1.36-15.40) | **0.01** | **n.d.*** |  |
| RF IgM positive, % | 73% | 65% | 1.49 (0.48-4.65) | 0.49 |  |  |
| Biological DMARDs (%) | 38% | 35% | 1.14 (0.39-3.34) | 0.82 |  |  |
| Randomization arm 3 (Taper) (%) | 50% | 35% | 1.82 (0.63-5.27) | 0.27 |  |  |

* Multivariable model not done (n.d.) as only one variable within each model subsection was significantly associated with the outcome.

Final RETRO-specific multivariable model including swollen joint count-28 and anti-CCP2 positivity had a pseudo-R^2^ of 15%. Final model including predictors common to both cohorts (DAS at baseline, anti-CCP2 positivity, gender) had a pseudo R^2^ of 9% in the RETRO study. Choice of these common predictors was based on known predictors of flare reported in literature as well as commonalities in predictors for both cohorts, reported above.

**Table S2**: Inflammatory parameters and baseline demographics separated by low/high circulating calprotectin levels (ng/mL) split on its median at the moment of DMARD tapering/stop. P-values are based on t-tests, Mann-Whitney tests, or Chi-square tests for the differences in mean, median, and frequency of reported variables, respectively.

| IMPROVED study (n=104) | Low calprotectin (n=52) | High calprotectin (n=52) | p-value |
| --- | --- | --- | --- |
| DAS44 at tapering moment, mean (SD) | 0.8 (0.4) | 0.9 (0.4) | 0.22 |
| ESR, median (IQR) | 6 (3-9) | 8 (3-15) | 0.38 |
| CRP, median (IQR) | 3 (2-3) | 6 (3-8) | **0.03** |
| VASgh, median (IQR) | 6 (2-25) | 6 (2-19) | 0.73 |
| Tender joint count-28, median (IQR), (minimum-maximum) | 0 (0-0), (0-3) | 0 (0-0), (0-4) | 0.49 |
| Swollen joint count-28, median (IQR),  (minimum-maximum) | 0 (0-0), (0-1) | 0 (0-0), (0-3) | 0.53 |
| Age, mean years (SD) | 49 (14) | 49 (12) | 0.95 |
| Female, % | 62% | 67% | 0.54 |
| BMI, mean (SD) | 25 (3) | 26 (4) | 0.09 |
| Ever smoker, % | 42% | 49% | 0.49 |
| Disease duration, weeks median (IQR) | 19 (10-30) | 19 (9-50) | 0.63 |
| Anti-CCP2 IgG positive, % | 81% | 84% | 0.64 |
| RF IgM positive, % | 78% | 80% | 0.73 |
|  |  |  |  |
| RETRO study (n=57) | **Low calprotectin (n=29)** | **High calprotectin (n=28)** | **p-value** |
| DAS28-ESR at tapering/stop moment, mean (SD) | 1.7 (0.9) | 1.8 (0.6) | 0.67 |
| ESR, median (IQR) | 11 (5-17) | 14 (10-19) | 0.16 |
| CRP, median (IQR) | 28 (11-35) | 29 (13-59) | 0.36 |
| VASgh, median (IQR) | 1 (0-8) | 0 (0-1.5) | 0.10 |
| Tender joint count-28, median (IQR), (minimum-maximum) | 0 (0-0), (0-1) | 0 (0-0), (0-0) | **0.02** |
| Swollen joint count-28, median (IQR),  (minimum-maximum) | 0 (0-0), (0-2) | 0 (0-0), (0-1) | 1.00 |
| Age, mean years (SD) | 53 (12) | 59 (5) | 0.08 |
| Female, % | 66% | 64% | 0.92 |
| BMI, mean (SD) | 26 (5) | 25 (3) | 0.39 |
| Ever smoker, % | 38% | 25% | 0.29 |
| Disease duration, years median (IQR) | 5 (3-10) | 6 (2-10) | 0.72 |
| Anti-CCP2 IgG positive, % | 57% | 66% | 0.47 |
| RF IgM positive, % | 66% | 71% | 0.63 |
| Biological DMARDs (%) | 34% | 43% | 0.52 |
| Randomization arm 3 (Stop) (%) | 38% | 46% | 0.52 |
